# Detection of cancer cells in the cerebrospinal fluid: current methods and future directions

**DOI:** 10.1186/2045-8118-8-14

**Published:** 2011-03-03

**Authors:** Cody L Weston, Michael J Glantz, James R Connor

**Affiliations:** 1 Department of Neurosurgery (H110), Penn State University M.S. Hershey Medical Center, 500 University Dr., Hershey, PA, 17033-0850, USA

## Abstract

The spread of cancer into the central nervous system is a serious problem leading to neurological symptoms and rapid mortality. The current tools available for detecting the spread of cancer into the cerebrospinal fluid (CSF) are cytology, neurologic examination, and neuroimaging. All three of these methods can be applied in concert to reach a diagnosis, but they all suffer from a lack of sensitivity, leading to delays in treatment in many cases. An overview of research tools in the field of CSF cancer detection reveals a variety of promising technologies that can be used to answer questions about the biology of metastatic cancer and to develop more powerful clinical detection methods. Methods currently under investigation include new immunocytochemistry methods and flow cytometry for the *in vitro *detection of cells. Additionally, polymerase chain reaction, fluorescence *in situ *hybridization, capillary electrophoresis with laser-induced fluorescence, and mass spectrometry using matrix-assisted laser absorption-deionization time-of-flight and surface-enhanced laser desorption/ionization time-of-flight techniques are being tested for *in vitro *assessment of the non-cellular biomarkers in CSF. For *in vivo *detection of cancer in the CSF, research techniques include certain quantum dot platforms as well as magnetic iron oxide nanoparticles. As systemic therapies for cancer improve, the CNS is becoming a more common site of disease recurrence. This increases the importance of effective detection methods in the CSF, since early intervention can maximize therapeutic benefit. Furthermore, many cell-based detection methods can be combined with therapeutic agents to serve multiple medical functions through a common targeting system.

## Review

### Introduction

The ability to detect and characterize malignant cells in the CSF derived from primary neural and extraneural cancers may allow us to answer fundamental questions about the biology of metastatic spread through identification and characterization of the cancer cell populations capable of infiltrating the CSF. Cancer may reach the CSF through hematogenous spread, direct extension from the tumor itself, or by migration along perineural or perivascular spaces [1,2]. Improved detection of CSF malignancy is a clinical imperative as well, since current diagnostic techniques are insensitive, resulting in a delay in diagnosis until disease-related symptoms are profound and irreversible, and therapeutic options are limited or non-existent. Cancer cells infiltrating the CSF can lead to neoplastic meningitis, a rapidly progressive and fatal condition characterized by serious neurologic deficits [3]. Across all cancer diagnoses, leptomeningeal involvement is seen in roughly 5% of patients, and carries a poor prognosis with median survival under 3 months characterized by rapid neurologic decline [3,4]. The biochemical and molecular mechanisms underlying this process remain unknown. Their elucidation may dramatically improve our ability to predict, treat, and even prevent this increasingly frequent and uniformly fatal complication of cancer. The primary aim of this review is to describe the state of the field of cancer cell detection in the CSF with the goal of inspiring translational scientists to develop the next generation of clinical detection strategies.

### Current clinical techniques

A prior review by Chamberlain and colleagues described the three primary methods of detecting leptomeningeal metastasis and the spread of tumor cells into the CSF: CSF cytology, neurologic examination, and radiographic imaging of the neuraxis [5]. While all three of these techniques can be used to diagnose leptomeningeal metastases, they all suffer from limitations and generally only detect the presence of tumor late in the disease course.

CSF cytology, in which CSF is prepared and examined under a microscope to look for cells, is currently considered the gold standard for diagnosis of leptomeningeal metastasis [5]. While it is highly specific (> 95%), it suffers from a lack of sensitivity (< 50%) [5]. However, optimum technique including obtaining 10.5 mL or more of sample, performing a second collection if the first is negative, taking CSF from as close to the tumor as possible (when the tumor is visible with neuroimaging) and expeditious processing, can maximize the likelihood of detecting cells when they are present in the CSF [6]. In all the studies discussed that compare another method to cytology, it is important to consider that proper handling procedures like those above could improve the diagnostic efficiency of cytology. Therefore, the methods used to prepare CSF for cytology and other analyses should be considered for all scientific and clinical investigations involving CSF cells. Even with this approach, however, false-negative results and delays in diagnosis are still a major concern given the need to direct these patients to early aggressive treatment [6,7]. Immunostaining has been used to improve detection, identify the original tumor type and guide management, suggesting that better immunostaining and cell labeling techniques could be used in the clinic as well as for answering scientific questions about cancer [7,8]. For instance, in the field of neuro-oncology, there is a hope that understanding the phenotypic changes (through surface marker expression and genetic mutations) that encourage metastasis will reveal new drug targets for the containment and elimination of cancers. One major weakness of CSF cytology is that uniform distribution of cancer cells in the CSF is rarely a safe assumption. For example, a 1998 study by Chamberlain showed that 14 of the 30 patients with leptomeningeal metastasis tested were found to have blockages in CSF flow [9]. Furthermore, in 90 taps on 52 patients comparing lumbar and ventricular shunt taps, there were 12 cases of positive CSF cytology in lumbar taps with negative shunt taps, whereas there were only 2 cases with positive shunt taps and negative lumbar taps [10]. Even when blockages are not present in the CSF, malignant cells will be found in greater concentration nearer to regions of the CNS exhibiting radiographic or clinical signs due to the diffusion of cells [6,11]. These latter examples emphasize the point made earlier that collecting CSF most proximal to the tumor is likely to be the most informative, an intuitive but important point because CSF samples are generally only withdrawn from one site. Although cytology is considered the gold standard for detection of neoplastic cells in the CSF, its low sensitivity makes the interpretation of other measures difficult. When a method is shown to be more sensitive than CSF cytology, it should be noted that it is difficult to distinguish between false positives and true increases in sensitivity without a demonstrated association with clinical outcome.

The second standard method of detecting cancer in the CSF is by clinical findings including headache, mental status changes, diplopia, weakness, and sensory loss [1]. This method is challenging because many patients with CSF spread of neoplastic cells have concomitant primary tumors, surgeries, and radio- and chemotherapies that can confound detection by causing abnormal findings on neurologic examination. Additionally, tumor growth sufficient to manifest clinical signs and symptoms is often a late finding in the course of CSF spread, rendering any potential treatments less effective. Finally, while appropriate treatment may slow or arrest tumor progression, established neurologic deficits are rarely reversible. For all these reasons, it is imprudent to wait for clinical manifestations to appear before initiating treatment and emphasizes the need for a reliable detection method.

The third primary means of detecting cancer in the CSF is through neuroimaging. Among the neuroimaging modalities, gadolinium-enhanced MRI of the neuraxis is the method of choice [1]. MRI has the potential to detect the presence of cancer in the leptomeninges when cytologies are negative, and is more likely to detect solid tumor metastases than hematologic metastases to the CSF [12,13]. Gadolinium-enhanced MRI may be more sensitive than cytology, but its lower specificity precludes it from replacing cytology as the gold standard for diagnosis [14]. Also, the clinical use of MRI to aid diagnosis has not shown any clear survival benefit [3]. This lack of improvement in survival is probably due to both the limited spatial resolution of MRI and the paucity of effective treatments for CSF infiltration of cancer. While MRI is an important clinical tool for detecting leptomeningeal metastasis, its limitations highlight how emerging technologies could fill gaps in the clinical armamentarium. To maximize the chances of successfully eliminating cancer in the CSF, it will be necessary to detect changes at the cellular level rather than waiting for a macroscopic tumor to emerge.

### Emerging research directions

Because the clinically utilized methods for detecting the presence of cancer cells in the CSF suffer from multiple limitations, a wide array of alternatives are being developed to better answer clinical and basic science questions. Broadly, these efforts focus on both improved *in vitro *analysis of CSF and *in vivo *imaging and spectroscopy techniques.

### In vitro assays

#### Immunocytochemistry

As far back as 1984, the use of monoclonal antibodies like UJ13A for neuroectodermal cells, LE61 for cytokeratin, and 2D1 for leucocytes to detect and diagnose cancer in the CSF, showed advantages over conventional cytology [15]. Combined with the vast array of antibodies now available commercially, effective targeting of metastatic and primary CNS cancer cells in the CSF should already be possible for most tumor types [16-18]. Promising antibodies and targeting peptides abound, with new discoveries occurring rapidly. While not intended to be exhaustive, Table 1 shows a few examples of promising ligands for immunocytochemical applications. The vast majority of these targeting ligands have not been evaluated for their utility in the CSF, so much work remains to establish them as viable detection tools for leptomeningeal metastasis. In the blood, there is an increasing interest in identifying circulating tumor cells (CTCs) using antibody and molecule tagging. These methods, including the FDA-approved CellSearch technology, should be investigated in the CSF, particularly in primary CNS tumors where the CSF may be seeded before the blood [19,20].

**Table 1:** Selected examples of promising molecular markers for targeted detection

Disease	Marker	Source
Glioma	IL-13	[76]

Breast Cancer	HER2	[22]

Lung Cancer	HER2	[77]

Malignant Melanoma	9.2.27	[68]

Genitourinary Tumors (Ovarian)	HER2	[77]

Head and Neck Cancers	EGFR	[78]

Leukemia	CD20, CD52	[79]

Lymphoma	CD20, CD52	[80]

The arrival of quantum dots coated for biomedical applications using polymer coating, lipid encapsulation, or ligand exchange provides an ideal fluorescent tag for identifying cancer cells *in vitro *[21-23]. Organic fluorophores such as fluoresceins and rhodamines are limited by low photostability, narrow absorption and emission spectra, and comparatively low quantum yields [24]. In contrast, quantum dots are photostable, have broad absorption and narrow emission spectra, and high quantum yields. Quantum dots are also preferable to conventional fluorophores for cancer cell detection because they have also been shown to specifically label cells and subcellular targets such as breast-derived cancer cells when combined with streptavidin/biotin binding to HER2 [22]. Outside the CSF, HER2-targeted quantum dots have been used to stain tumor specimens, resulting in a promising quantitative prognostic marker in breast cancer [25].

Quantum dots are now proving successful at improving cellular detection using a variety of antibody and peptide ligands [26]. As new conjugation techniques such as DNA-bridged conjugation emerge, the efficiency of attaching targeting ligands is continuing to improve [27]. When these DNA-bridged quantum dot conjugates were used in microfluidic detection arrays, quantum dots that were conjugated to targeting molecules were four orders of magnitude more sensitive than similar assays using conventional fluorophores. More importantly, the detection sensitivity of these agents exceeded the other methods of quantum dot targeting that they tested by at least four fold. Therefore, emerging technology continues to improve the detecting ability of quantum dot-based assays.

#### Flow Cytometry

Another promising method for the detection of cancer cells in the CSF is flow cytometry. This has been attempted mostly for hematologic tumors (leukemias and lymphomas) and has shown promise as a complementary method to cytology [28]. In work by Nuckel and colleagues for example, flow cytometry in patients with hematological malignancy identified 12 patients that had detectable cells by both modalities, 3 patients that had positive cytologies but no abnormal findings on flow cytometry, and 3 patients that had negative cytologies but abnormalities on flow cytometry [28]. Another example showed far more sensitivity: When CSF flow cytometry was performed with fluorophore tagging of CD antigens, CNS lymphoma was found in 22% of 51 cases compared to a detection rate of only about 2% for cytology, a statistically significant difference (*P *= 0.002). Impressively, flow cytometry required a neoplastic clone to make up only 0.2% of all CSF lymphocytes in order to be detected. For comparison, conventional cytology generally requires 5% tumor cells to find a positive result. The same study showed higher sensitivity in relapsed CNS lymphoma, although the sample size was smaller [29]. One concern with this report was the extremely low detection rate of leptomeningeal disease by cytology, suggesting an unusual patient sample, suboptimal handling of CSF, or a high number of false positive results from flow cytometry. An additional benefit of flow cytometry is that automated methods are available that allow the rapid acquisition and processing of diagnostic data, meaning that its use may not lead to significant time expenditures from the clinicians or researchers if it is used to supplement conventional cytology routinely [30]. More recently, a larger study examining 123 patients with B-cell non-Hodgkin's lymphoma found that flow cytometric analysis using a 9-antibody panel detected 27 positive cases (22%) while cytology only detected 7 cases with 3 suspicious cases [31]. In the Quijano report, only 1 with positive cytology did not have a corresponding positive flow cytometry result, and further analysis by immunocytochemistry ruled out CSF malignancy. Another recent study looked at clinical outcomes in patients with occult CNS involvement (detectable by flow cytometry but not conventional cytology) compared with patients that had positive cytologies and no detectable CNS involvement at all [32]. They found that positive flow cytometry results alone did not correlate with significantly higher overall survival, but that they did correlate significantly with a higher probability of CNS relapse (*P *= 0.05). This is encouraging for the future of this technique, since occult levels of neoplastic cells in the CSF appear to have a bearing on patient risk. The fact that only positive cytology showed a significant relationship with overall survival suggests a continuum of severity in which increased cell number corresponds to poorer prognosis and greater need for intervention.

One caveat to the use of flow cytometry on CSF samples, however, is that the inherently low cell number and suboptimal cell environment mean that cancer cells decay rapidly following removal from patient, even more so when centrifuged repeatedly [7]. Other potentially severe limitations of flow cytometry include a high rate of false positives at low cell counts (< 25 cells/μL) and a poor ability to provide differential data (poor differentiation between monocytes and eosinophils, and inability to detect mitoses and neoplastic cells) [33,34]. These considerations limit the current clinical utility of flow cytometry, but are not necessarily insurmountable by future developments. Many of the studies in the current literature suffer from methodological flaws in the CSF handling that may be limiting the effectiveness of the technology for this application. Implementation of more stringent handling guidelines with respect to speed of processing, judicious use of centrifugation, use of a buffer to promote cell viability, and the use of more robust fluorescent tags may make this technology more useful than the earlier studies suggest [6,7]. Finally, the widespread use of flow cytometry for the detection of neoplastic cells in the CSF is limited at this point by variations in the equipment and methods. The implementation of standardized protocols across clinical labs will be necessary before flow cytometry can be implemented in a routine way alongside conventional cytology [7].

#### Polymerase chain reaction/genetic detection

One method that has been used to identify genetic changes in cancer cells of the CSF is Fluorescence *In-Situ *Hybridization (FISH). In one study, 13 of 15 CSF samples with cells from leptomeningeal cancer patients showed numerical chromosomal abnormalities compared to no chromosomal abnormalities in the 10 control samples [35]. The study was limited by the use of patients that had already been diagnosed with cytology, meaning that FISH was in fact less sensitive than cytology. In addition, 7 samples had lysed cells or no cells in this study. While some of those unusable samples were probably always devoid of useful cells, the implementation of the handling guidelines mentioned above may have yielded more useable cells for analysis [6]. Specifically, assurance of fast sample processing would be straightforward to implement and would increase the likelihood of detecting cells if they were originally present in the sample. While these guidelines had not been published at the time of van Oostenbrugge and colleagues' investigation, their results serve to demonstrate how future adherence to handling guidelines can improve data yield during CSF analyses.

In another notable study, FISH was performed on tumor samples and CSF cytology slides of 16 patients with metastatic breast cancer to the leptomeninges to compare the primary tumor's HER2 status to that of the metastatic cells [36]. Using this method, they were able to show concordance in HER2 amplification between primary tumor and CSF metastatic cancer cells in all but 2 of the 16 cases. In the discordant cases, the primary tumor was positive for HER2 by immunohistochemistry but the CSF cells were negative for HER2 amplification by FISH. Because the HER2 receptor can be targeted for detection and therapy, this information could be applied clinically [36]. It should be noted that FISH is being used to detect chromosomal abnormalities, and would therefore be unable to distinguish between a malignant change and an increase in copy number unrelated to malignancy. Therefore, a genetic aberration would have to be strongly associated with malignancy for this technique to be useful. Although its sensitivity may preclude its use as a front-line screening tool, the existing literature does suggest that it could mature into a useful tool for guiding clinical decisions.

Another approach to improving the sensitivity of cell detection is to use polymerase chain reaction (PCR) methods to amplify DNA specific for cancer cells. One example is the amplification of IgH rearrangements in the CSF of patients with CNS lymphomas [37]. More commonly, reverse-transcription polymerase chain reaction (RT-PCR) is being investigated as a method for assaying the RNA found in CSF. It offers the advantage of amplifying small amounts of RNA into a much larger sample of cDNA; thus only a few neoplastic cells are needed in the CSF. Perhaps the biggest limitation of RT-PCR, however, is the need to have the appropriate primers for the type of RNA being assayed. This could limit the utility of this approach as a screening tool in cancers with heterogenous changes in RNA expression. Despite these concerns, RT-PCR has been used successfully to detect leptomeningeal metastasis from a variety of primary sources of cancer. For CNS involvement of lymphoma, RT-PCR using probes for clonal immunoglobulin gene rearrangements demonstrated positive amplifications in 5 of 13 CSF specimens that were considered negative for malignancy by cytology and 5 of 7 specimens that were only considered suspicious for lymphoma by conventional cytology [38]. However, only 2 of the 4 specimens with positive cytology had positive RT-PCR amplifications. In malignant melanoma, RT-PCR of CSF using primers for MART-1, MAGE-3 and tyrosinase (all known melanoma markers) were positive for at least one of the markers in 12 patients (vs. 1 positive cytology) of the 37 patients tested, and correlated significantly with development of CNS metastasis at 3 months [39]. More recently, RT-PCR for human mammoglobin has been shown to have greater sensitivity than cytology (can detect 1 malignant cell in 10^6 normal cells) and has been successfully used to diagnose leptomeningeal metastasis in a breast cancer patient [40]. For leptomeningeal involvement of leukemias and lymphomas, the technique of single-cell PCR analysis has potential as a detection method [41]. Able to distinguish between polyclonal reactive and monoclonal neoplastic increases in B-cells, single-cell PCR could successfully identify five of the six B-cell neoplasms as having monoclonal responses.

#### Noncellular biomarkers

In addition to detecting cancer cells in the CSF through direct methods, a considerable amount of effort has focused on indirectly detecting CSF malignancy through changes in expression patterns of proteins and metabolites in CSF samples. The proteomic/metabolomic approach could theoretically be more sensitive because even a few cells could alter the chemical milieu through disruption of the blood-brain barrier or through direct shedding of detectable molecules. Examples of CSF biomarkers for cancer are many. In an early study, carcinoembryonic antigen (CEA) and the β subunit of human chorionic gonadotropin (β-HCG) were found to correlate with leptomeningeal infiltration of the CSF by carcinoma [42]. Another early study found intrathecal synthesis of CEA in 89% of meningeal carcinomas and 47% of intraparenchymal carcinomas, suggesting a future role in detecting at least the cancers that communicate with the CSF [43]. This limits the use of CEA (and many CSF proteins) because solid parenchymal tumors could yield proteins that could be mistaken for evidence of leptomeningeal disease, resulting in unindicated and potentially harmful intrathecal treatments or neuraxis radiation. More recently, detection of tumor markers using a Modular Analytics Serum Work Area found intrathecal synthesis of CEA, CA125, CA15.3, and CA19.9 successfully differentiated 17 of 18 patients with leptomeningeal metastasis from 50 patients with other neurological diseases [44]. These investigators also found that VEGF Index was a relatively sensitive (83.3%) and specific (88.4%) means of detecting leptomeningeal metastasis. It is important to note that this study did not use other cancer controls such as solid CNS metastases, so further investigation of these markers would be necessary before clinical applications could be considered. Additional studies using High Performance Thin Layer Chromatography on CSF samples could differentiate medulloblastoma patients and astrocytoma patients from controls with a *P *< 0.0002 using ganglioside GD3 [45]. An important consideration in any study of CSF molecular biomarkers is that disease processes can disrupt the blood-CSF barrier, leading to infiltration of proteins. Neglecting this can lead to false assumptions about increased levels of intrathecally produced markers, so it is important to establish the state of the blood-CSF barrier using a measure such as albumen quotient [46].

Detection of amino acid profiles in the CSF using capillary electrophoresis followed by laser-induced fluorescence (CE-LIF) detection has been performed in 3 patients with acute lymphoblastic leukemia [47]. This study revealed significantly different amino acid "fingerprints" compared to reference values. Despite these promising findings, the study was limited by its small sample size and choice of patients who were being treated with high-dose methotrexate. Follow-up studies using the same methodology could not be found in the literature. CE-LIF has since been used for a variety of other applications ranging from amino acid detection in Huntington's disease to the analysis of wine, but it has been neglected in cancer applications [48]. Since CE-LIF methods require a small sample volume (50 μL) and a commercially available instrument, the technique seems ripe for further use in exploring amino acid biomarkers.

Mass spectrometry using matrix-assisted laser absorption-deionization time-of-flight (MALDI-TOF) and surface-enhanced laser desorption/ionization time-of-flight (SELDI-TOF) techniques has also been applied to the CSF to detect malignancy. Studies of MALDI-TOF and SELDI-TOF MS in serum have demonstrated high sensitivity and specificity for detecting protein biomarkers related to a variety of cancers including prostate and ovarian, and have been used in both blood and CSF [49-54]. CSF-focused work has used SELDI-TOF and MALDI-TOF analysis to identify promising protein biomarkers in inflammatory and neoplastic disease [55]. Another investigation used TOF mass spectrometry in concert with other proteomic methods (Two-dimensional gel electrophoresis and cleavable isotope-coded affinity tag analysis) to develop a 21-biomarker panel able to differentiate CSF samples according to astrocytoma grade [56]. A later study by Dekker and colleagues used MALDI-TOF MS of CSF samples and found sensitivity and specificity of 79% and 76% respectively when differentiating breast cancer without leptomeningeal metastasis from breast cancer with it, suggesting no clear benefit to the use of MALDI-TOF compared to current clinical detection methods [50]. Despite widespread use, SELDI-TOF and MALDI-TOF techniques have a variety of limitations. First of all, both methods are probably missing low-abundance proteins and peptides because the spectra of abundant proteins are masking them [57,58]. Fortunately, other proteomic methods are available for assessing these low-abundance proteins, such as isobaric tags for relative and absolute protein quantification (iTraq), which have yet to be applied to profiling CSF malignancy [59]. SELDI-TOF in particular carries caveats because of its sensitivity to procedural variation, an unclear relationship between peaks and known proteins and peptides involved with the disease process, and an inability to resolve molecules smaller than about 100 kDa, which excludes many biologically relevant peptides [60]. Reproducibility issues have been noted in several cases and limit the usefulness of both SELDI-TOF and MALDI-TOF without refinements in protocol [58,61]. The last issue is not a problem for MALDI-TOF, which can detect molecules as small as 1 kDa. These methods are also not ideal for particularly large molecules, which are better detected with electrophoresis and immunoblotting [62]. Despite these considerations, mass spectroscopy methods benefit from assessment of soluble markers, which are likely to be more abundant than cancer cells. The emphasis on molecular detection rather than cellular detection also lessens concerns associated with using frozen specimens, which may destroy or alter cells but have fewer effects on free molecules. MALDI-TOF is likely to continue to be useful in research, but much work remains to establish the reproducibility and validity of the spectroscopic profiles. Used with other proteomic tools, it should serve as a valuable part of the proteomic armamentarium for CSF studies.

Using another potentially useful approach, zymography for matrix metalloproteinases, particularly MMP2, and pMMP9 reportedly detect the presence of primary and metastatic brain tumors [63]. Interestingly, leukocyte counts were normal in these samples and MMP was still an effective strategy with high sensitivity and specificity. This is important because MMPs would be expected to accompany leukocytosis and inflammation, but their independent increase suggests that their role in angiogenesis or another process is responsible for their elevation.

Finally, trying to detect the fewest number of cells possible leads to a need either to improve sensitivity directly through more powerful targeting systems or to enrich target population of cells. While RT-PCR is one approach for amplifying rare molecules, another method that deserves some consideration for increasing the cell number is through culture of the CSF samples. While it may prove impossible to meet with routine success with this method, any successful cultures derived from CSF could help answer important questions about metastatic behavior. To date, only a few cases of successful isolation of cell lines have been documented, and their properties have not been examined through the lens of understanding the process of CSF metastasis [64,65]. Although these studies were not related directly to diagnostic use, they demonstrate that cells can be cultured from the CSF, at least in certain cases. Now that techniques have been established to isolate cancer progenitor cells from larger populations of cancer cells derived from gliomas, culturing these samples is especially important to answer questions about the diagnostic and prognostic value of these cells and their characteristics [66]. If methods to culture neoplastic cells from patient CSF could ever be refined to the point where growth was reasonably common, the growth properties of the cells in vitro could have useful prognostic implications.

### In vivo detection

For all their advantages, all *in vitro *approaches suffer from the collection limitations mentioned above. To be able to examine the entire CSF pool, *in vivo *methods are necessary (possibly with multiple access points in the event of an obstruction of flow due to solid tumor or other changes in the CNS anatomy). Therefore, multiple modalities of *in vivo *CSF screening for cancer are under development.

#### Magnetic iron oxide nanoparticles (MIONs)

Iron oxide, is a potentially useful targeted imaging agent because of its ability to function as a T2 contrast agent for MRI [67]. In cell culture, magnetic iron oxide nanoparticles (MIONs) have been demonstrated to specifically alter T2 properties of melanoma and breast cancer cells when conjugated to 9.2.27 and HER2, respectively [68]. Furthermore, MIONs have been successfully combined with quantum dots to allow for multimodal imaging [69].

#### Quantum Dots

The desirable luminescent properties of quantum dots have led to multiple investigations of their *in vivo *applications. In animal models, quantum dots have been successfully targeted to a variety of tumors [26]. The critical concern for human use, however, is that the most common quantum dot materials are toxic and they are not known to be safely eliminated from the body [21]. Recently, silicon-based quantum dots have been engineered and successfully used *in vivo *as part of multimodal diagnostic and therapeutic agents targeted to tumors with antibodies and peptide ligands. To create an imaging platform of this nature, Erogbogbo and colleagues combined quantum dots with magnetic iron oxide nanoparticles and packed the combination into micelles for *in vivo *delivery. Combining both particles in one platform allowed for both optical and magnetic resonance imaging using targeted agents [70].

## Conclusions

As therapies for extra-neural cancers become more successful, the CNS in general and the CSF in particular are increasingly becoming the most common sites of disease recurrence [71-73]. Emerging cell tagging methods show much promise for *in vitro *assessments and *in vivo *surveillance, although further studies must be done to establish their true clinical potential. In addition to the methods under investigation, the literature appears to lack any published efforts in some other potentially useful techniques, including multiplexed cytokine analysis, which has been useful in neurodegenerative diseases such as ALS, and magnetic resonance spectroscopy, which is a common method of assessing neural biomarkers noninvasively [74,75].

The ultimate goal of diagnostics in CSF is to detect the earliest meaningful sign of neoplastic presence in the CSF to enable clinicians to react with the most relevant therapy. Currently, three key methods exist that are fairly specific but lack the sensitivity early in the disease course that will enable the most timely therapeutic response. Future therapies must overcome the challenges of detecting few cells in a comparatively large space either directly or by profiling soluble biomarkers that herald their arrival. In the past decade, advances in clinical imaging and targeting ligand development have improved our ability to detect leptomeningeal involvement. Based on the state of the field, flow cytometry appears the most promising among the emerging technologies detailed here, particularly for aiding in the diagnosis of hematologic malignancies. Profiling of the soluble markers of the CSF is likely to become important in the future, but it is too early to say which method will prove most reliable in attaining that end.

With the CSF more clinically relevant than ever, methods for surveillance are becoming increasingly important [2]. The effective management of cancer in the CSF will begin with the development of more sensitive diagnostic tools. With focused honing of these techniques, the clinical armamentarium will expand and empower clinicians to relieve patients of debilitating neurological symptoms and deadly disease progression with fewer delays and greater confidence.

## Competing interests

The authors declare that they have no competing interests.

## Authors' contributions

CLW drafted the manuscript, performed revisions, and provided the final manuscript draft. MJG provided clinical perspective on the article, offered criticism, provided revisions, and gave final approval to the submitted manuscript. JRC established the concept of the article, offered criticism, provided revisions, and gave final approval to the submitted manuscript. All authors have read and approved the final version of the manuscript.
